# *In vitro* infection with classical swine fever virus inhibits the transcription of immune response genes

**DOI:** 10.1186/1743-422X-9-175

**Published:** 2012-08-28

**Authors:** Li Feng, Xiao-Quan Li, Xiao-ning Li, Jun Li, Xian-Ming Meng, Hong-Yun Zhang, Jing-Jing Liang, Hui Li, Shi-Kai Sun, Xin-Bin Cai, Li-Juan Su, Shan Yin, Yan-Sheng Li, Ting Rong Luo

**Affiliations:** 1 State Key Laboratory for Conservation and Utilization of Subtropical Agro-bioresources, Guangxi University, 100 Daxue Road, Nanning, 530004, Guangxi, China; 2 College of Animal Sciences and Veterinary Medicine, Guangxi University, 100 Daxue Road, Nanning, 530004, Guangxi, China

**Keywords:** Classical swine fever virus (CSFV), Immune response genes, Real-time RT-PCR

## Abstract

**Background:**

Classical swine fever virus (CSFV) can evade the immune response and establish chronic infection under natural and experimental conditions. Some genes related to antigen processing and presentation and to cytokine regulation are known to be involved in this response, but the precise mechanism through which each gene responds to CSFV infection remains unclear.

**Results:**

In this study, the amplification standard curve and corresponding linear regression equations for the genes SLA-2, TAP1, SLA-DR, Ii, CD40, CD80, CD86, IFN-α, and IFN-β were established successfully. Real-time RT-PCR was used to quantify the immune response gene transcription in PK-15 cells post CSFV infection. Results showed that: (1) immune response genes were generally down-regulated as a result of CSFV infection, and (2) the expression of SLA-2, SLA-DR, Ii and CD80 was significantly decreased (p<0.001).

**Conclusion:**

We conclude that in vitro infection with CSFV inhibits the transcription of host immune response genes. These findings may facilitate the development of effective strategies for controlling CSF.

## Background

Classical swine fever (CSF) is a devastating disease, which is responsible for substantial economic losses due to the death of valuable livestock. For this reason, classical swine fever virus (CSFV), the causative agent of CSF, is a listed disease by the World Organisation for Animal Health [1,2]. Since the implementation of strict immunization measures, changes have been reported in the epidemiological form and clinical signs of CSF. Large-scale outbreaks are now rare, but sporadic epizootics still occur frequently with chronic, atypical forms of the disease. Abortions, stillbirths, mummifications, malformations, and the birth of weak piglets have also been observed [3]. Although some countries, such as Australia and Japan, have succeeded in eradicating the disease, CSF is still epidemic or endemic in many countries in Europe and Asia, including China [4].

CSFV is a single positive strand RNA virus of the genus *Pestivirus* in the family *Flaviviridae*, approximately 12.3 kilobases (kb) in length [5-8]. The virus has been detected in semen, gonads, tonsils, and lymph nodes, even in vaccinated pigs [9,10]. It has been shown to evade the host immune system and to establish chronic infection under both natural and experimental conditions [11]. Specifically for CSFV infection, host anti-viral Type I IFN, such as alpha-interferon (IFN-α), was suppressed in infected dendritic cells, and some other host cytokines including interleukin (IL)-6, IL-10, IL-12, and TNF-α were also not induced [12]. There was an initial, short-lived increase in the transcription level of genes encoding for pro-inflammatory cytokines IL-1, IL-6, and IL-8 at 3 h, followed by a second, more sustained, increase at 24 h post CSFV infection [13]. Transcription levels for genes encoding for coagulation factors, tissue factors, and vascular endothelial cell growth factor (VEGF), all of which are involved in endothelial cell permeability, were also increased [13]. Sánchez-Cordón also reported that CSFV strain Alfort/187 from a Swiss isolate induced a change in cytokine expression (IL-2, IL-4, and IFN-γ) by the T-lymphocyte population (CD3^+^, CD4^+^, and CD8^+^) in serum, thymus and spleen.

In 2001, two independent studies confirmed that non-cytopathic bovine viral diarrhea virus (BVDV) can establish persistent infection by inhibiting the induction of type I interferon *in vitro* and *in vivo*[14,15]. However, in addition to evading the immune response by altering cell surface markers and cytokine induction, many viruses have developed the ability to infect immune cells -- a strategy which plays a key role in orchestrating the inhibition of antiviral immune responses. For example, the HIV-1 virus infects CD4^+^ T cells, and the depletion of these cells is a hallmark of HIV-induced AIDS. Previous studies have demonstrated that CSFV has a particular affinity for cells of the immune system, and can compromise the host immune response. During CSF, both B- and T-lymphocytes are depleted [16-18]. CD4^+^ and CD8^high+^ T- lymphocyte levels drop dramatically before the onset of viremia [19,20]. In addition, CSFV infection can suppress the function of T-lymphocytes isolated from CSFV-infected pigs [21,22]. CSFV exhibits an especially high affinity for phagocytes of the macrophage and monocyte lineage (reticulo-endothelial cells), mostly in the vascular endothelium [23,24]. Infection of these cells in the endothelium leads to an increase in vascular permeability, lymphopenia, thrombocytopenia, coagulation disorders, and atrophy of the thymus and bone marrow [25-27]. During the later stages of CSFV infection, peripheral monocytes, lymphocytes, and granulocytes can also carry viral antigens [28,29]. Further studies have revealed that CSFV induces apoptosis in lymphocytes and in neutrophil-lineage cells of the bone marrow [30-33].

Studies of the interactions between hosts and specific viruses are critical for understanding pathogenic mechanisms and the immune response to infection. CSFV shows a predilection for cells of the immune system and may alter transcription of immune response genes. The global transcriptional profiles of peripheral blood mononuclear cells during CSFV infection have shown that cellular genes present a low level of up- and down-regulation *in vivo*[34]. In order to gain more insight into the mechanisms of how CSFV affects host genes associated with immune response, we have investigated *in vitro* the activity of cellular genes in response to CSFV infection using the real-time RT-PCR.

## Results

### Real-time PCR determination of replication of CSFV genomic RNA in infected cells

**Figure 1:**
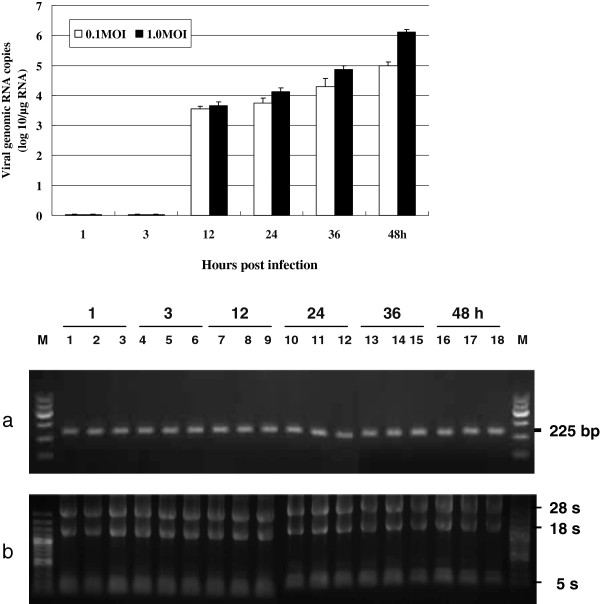
**Detection of CSFV on the infected PK-15 cells. Figure 1–1** CSFV genomic RNAs present in PK-15 cells at 1, 3, 12, 24, 36, and 48 h post-inoculation with different MOI of CSFV strain Shimen. Viral genome copy number was assessed by real-time RT-PCR. **Figure 1–2** RNA from PK-15 cells inoculated with the Shimen strain of CSFV. (**a**) GAPDH gene detection from PK-15 cells inoculated with Shimen strain of CSFV; (**b**) Total RNA detection from PK-15 cells inoculated with Shimen strain of CSFV. Lanes 1, 4, 7, 10, 13, and 16 show the mock PK-15 cells cultures at 1, 3, 12, 24, 36, and 48 h post-inoculation with CSFV. Lanes 2, 5, 8, 11, 14, and 17 show PK-15 cells culture inoculated with 0.1 MOI of CSFV at 1, 3, 12, 24, 36, and 48 h. Lanes 3, 6, 9, 12, 15, and 18 show PK-15 cells inoculated with 1.0 MOI of CSFV at 1, 3, 12, 24, 36, and 48 h.

To characterize the replication of CSFV, PK-15 cells were infected with the Shimen strain of CSFV, and then collected at 8, 12, 24, 36 and 48 h post-inoculation (hpi). The resulting CSFV-specific RNA loads in PK-15 cells at different times are shown in Figure 1. Infection with CSFV at an MOI of 0.1 resulted in 10^3.55±0.09^, 10^3.749±0.16^, 10^4.28±0.28^, and 10^5.00±0.11^ copies/μg RNA at 12, 24, 36, and 48 hpi, respectively. The corresponding numbers for an MOI of 1.0 are 10^3.66±0.14^, 10^4.12±0.13^, 10^4.86±0.12^, and 10^6.10±0.09^ copies/μg RNA at 12, 24, 36, and 48 hpi, respectively, thus demonstrating a significant dependence of CSFV replication in PK-15 cells on MOI, especially at the later time points. CSFV genomic RNA was not detected in PK-15 cell cultures at 1 and 3 hpi or in mock-infected PK-15 cells at any time point. Thus these differences were due only to the different MOI input, because total RNA extracted from all of the CSFV-infected PK-15 cells and mock-infected PK-15 cells was intact; characteristic bands of the 28s, 18s, and 5s RNA were observed (Figure 1b), and CSFV-infected and mock-infected PK-15 cells had similar expression of glyceraldehyde 3-phosphate dehydrogenase(GAPDH) (Figure 1a).

### CSFV infection down-regulates many host immune response genes

Transcriptional levels of immune response genes were identified in PK-15 cells based on (i) detection of mRNA levels coding for the CSFV E2 protein and CSFV genomic RNA, and (ii) identification of three characteristic bands of 28s, 18s, and 5s RNA from the total RNA extracted from CSFV-infected cells, as well as mock-infected cells, and the regular expression of the housekeeping gene GAPDH.

**Figure 2:**
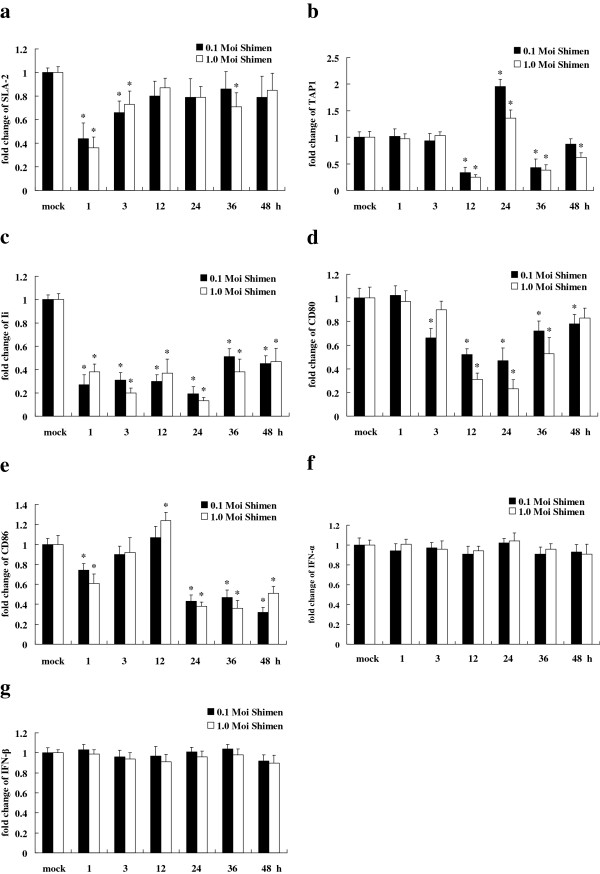
**mRNA changes in immune response genes following CSFV infection in PK-15 cells.** Transcription changes (**a** to **g**) of SLA-2, TAP1, Ii, CD80, CD86, IFN-α and IFN-β genes in PK-15 cells following CSFV infection with either a MOI of 0.1 or 1.0 at different time points of 1, 3, 12, 24, 36 and 48 hpi.

Tests were performed to investigate whether there was any correlation between infection of PK-15 cells with CSFV and changes in the mRNA expression of host immune genes associated with the MHC antigen presentation pathway, such as MHC class I swine leukocyte antigen 2 (SLA-2), MHC class II swine leukocyte antigen DR (SLA-DR) and MHC class II-associated invariant chain (Ii), co-stimulation molecules (CD40, CD80, CD86), or host anti-viral Type I IFNs (IFN-α, IFN-β). mRNA expression measured at various time points (1, 3, 12, 24, 36 and 48 hpi) by real-time RT-PCR showed that infection of PK-15 cells with CSFV at either a MOI of 0.1 or 1.0, resulted in down-regulation of seven immune response genes, namely SLA-2, TAP1, SLA-DR, Ii, CD40, CD80, CD86. Notably, some genes were up-regulated at later time points – TAP1 was slightly upregulated at 24 hpi, and CD86 at 12 hpi (Figure 2). In the MHC class I antigen presentation pathway, MHC class I molecules bind to endogenous peptides, which are then delivered from the cytosol into the endoplasmic reticulum by the transporter associated with antigen processing (TAP). Comparied to mock-infected cells at 3 hpi, MHC class I molecule SLA-2 was down-regulated during CSFV infection by 2.27-fold with a MOI of 0.1 and 2.78-fold with a MOI of 1.0 in infected PK-15 cells as early as 1 hpi. This downregulation was then slightly reduced to 1.52-fold with a MOI of 0.1 and 1.73-fold with a MOI of 1.0. TAP1 was expressed at low levels during CSFV infection except at 24 hpi, when it exhibited a slight increase by 1.96-fold with a MOI of 0.1, and 1.36-fold with a MOI of 1.0. Expression of anti-viral genes, IFN-α and IFN-β, remained unchanged during CSFV infection. In contrast, expression of both Type I IFNs was increased in PK-15 cells infected with an optimal dose of Newcastle disease virus, or transfected with poly I:C (data not shown), which served as positive controls for induction of a normal host anti-viral Type I IFN response. This result indicates that CSFV suppressed the up-regulation of the anti-viral Type I IFNs in PK-15 cells.

**Figure 3:**
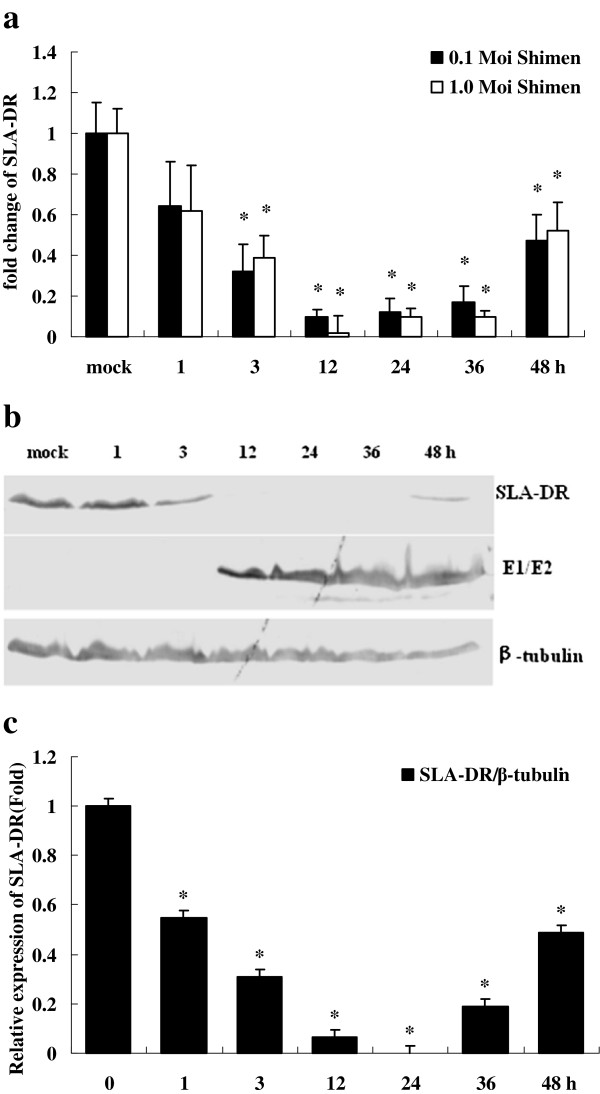
**Comparison of deferentially expressed SLA-DR by real-time PCR and Western blot analysis.** (**a**) mRNA changes of SLA-DR gene in PK-15 cells following CSFV infection at 1, 3, 12, 24, 36, and 48 hpi. (Note: Bars above columns represent standard deviations. * represents the significant difference (p<0.001) in comparison with the control by t-test). (**b**) Detection of changes in protein expression by western blot with specific antibodies against SLA-DR, β-tubulin, and CSFV glycoprotein E2 in PK-15 cells following CSFV infection at 1, 3, 12, 24, 36, and 48 hpi. The dimer of E1 and E2 (E1/E2) was detected with specific antibody against CSFV glycoprotein E2. (**c**) The quantification of changes in protein expression by the former western blot. The relative expression levels of SLA-DR were compared with that of β-tubulin. (Note: Bars above columns represent standard deviations. * represents the significant difference (p<0.001) in comparison with the control by t-test).

Overall, these results show that CSFV can induce transcriptional inhibition of important immune response genes. Functional molecules associated with exogenous antigen processing and presentation, such as MHC class II molecule SLA-DR and Ii, were transcribed at low levels during CSFV infection. SLA-DR and Ii genes were decreased notably as early as 1 h and remained at low levels until 48 h after CSFV-infection of both 0.1 and 1.0 MOI. Moreover, the lowest value of SLA-DR showed a 50-fold decrease at 12 hpi with infection of 1.0 MOI., whilst the values of SLA-DR displayed about 10-fold decline at 12 and 24 hpi with infection of 0.1 MOI as well as at 24 hpi with 1.0 MOI (Figure 3).

### Protein expression of SLA-DR and CD40 during CSFV infection

To determine the accuracy of the transcriptional data obtained by real-time RT-PCR, Western blot analysis was performed to detect expression of SLA-DR and CD40 in PK-15 cells infected with CSFV. It was found that SLA-DR expression was partially inhibited at 3 hpi and inhibited completely from 12–36 hpi. Expression of SLA-DR was detectible but very low at 48 hpi, whereas the E1/E2 glycoprotein of CSFV began to emerge at 12 hpi. Transcription of SLA-DR in PK-15 cells infected with CSFV was largely consistent with protein levels (Figure 3).

**Figure 4:**
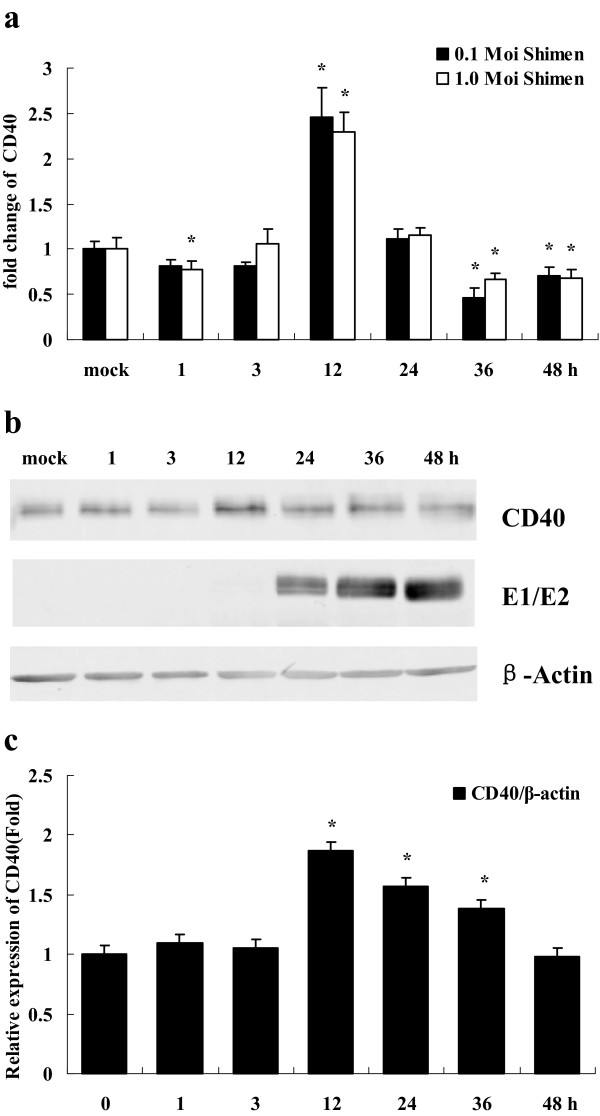
**Comparison of deferentially expressed CD40 using real-time PCR and Western blot.** (**a**) mRNA changes of CD40 gene in PK-15 cells following CSFV infection at 1, 3, 12, 24, 36, and 48 hpi. (Note: Bars above columns represent standard deviations. * represents the significant difference (p<0.001) in comparison with the control by t-test). (**b**) Detection of changes in protein expression by western blot with specific antibodies against CD40, β-tubulin, and CSFV glycoprotein E2 in PK-15 cells following CSFV infection at 1, 3, 12, 24, 36, and 48 hpi. The dimer of E1 and E2 (E1/E2) was detected with specific antibody against CSFV glycoprotein E2. (**c**) The quantification of changes in protein expression by the former western blot. The relative expression levels of CD40 were compared with that of β-tubulin. (Note: Bars above columns represent standard deviations. * represents the significant difference (p<0.001) in comparison with the control by t-test).

Relative to mock cells, the mRNA level of CD40 was inhibited in PK-15 cells infected with 0.1 or 1.0 MOI of CSFV at 1 hpi and with 0.1 MOI of CSFV at 3 h, but it was increased 2.5- or 2.3-fold, respectively at 12 hpi with 0.1 or 1.0 MOI of CSFV. Then the mRNA levels of CD40 were down-regulated at the last two time points of 36 or 48 hpi. Expression of CD40 protein in PK-15 cells infected with CSFV determined by Western blot analysis was basically consistent with corresponding changes in mRNA levels (Figure 4a, 4b).

## Discussion

Although the immune system plays a crucial role in the response to viral infection, viruses have evolved a number of strategies to interfere with host innate and adaptive immune functions. CSFV causes a range of acute and chronic diseases in pigs through both viral virulence and host factors [35]; the myeloid cell population, including monocytes, macrophages, and dendritic cells, are the early target cells of infection. CSFV has developed a mechanism to prevent the antiviral response, enabling the virus to use these migrating cells as transporters for delivery to different sites within the host, and the immunocytes and somatocytes are simultaneously damaged during CSFV infection.

We have used real-time RT-PCR to detect transcriptional changes of eleven genes, including the housekeeping genes GAPDH and 5′-UTR, of the CSFV genome in PK-15 cells *in vitro*. The primer sets and reaction conditions, including annealing temperature, T_m_, and plate-reading temperature, were optimized for each gene (Table 1). These primer sets were optimally designed because they resulted in a single product, distinguished by the production of a single melt-curve peak at all concentrations, and the production of a standard curve with an acceptable R^2^ (>0.99) (Additional file 1: Figure S 1). Real-time RT-PCR results were reproducible for each gene, and this improved method for detecting reliable gene mRNA messages has solved the common problem plaguing PCR of non-predicted amplification products.

**Table 1:** Primers used for real-time RT-PCR

**Gene**	**GenBank accession No**	**Function**	**Primer sequence (5**^**′**^**→ 3**^**′**^** )**	**Anneal Temp (°C)**	**T**_**m**_**(°C)**	**Plate-reading Temp (°C)**	**Fragment (bp)**
SLA-2 (swine leukocyte antigen 2)	AF464005	Presents peptides of endogenous proteins to CD8^+^ cytotoxic T cells and stimulates cellular immune response.	cgcacagactttccgagtg (F)	60	85.9	83	110 (287–396)
			gtctggtcccaagtagcag (R)				
TAP1 (transporter associated with antigen processing 1)	DQ227989	Transports efficient peptides into the ER for binding to MHC class I molecules.	ccgaacaccaatgtacctcc (F)	60	86.8	84	235 (743–977)
			tccacctggcagtcatagac (R)				
SLA-DR (swine leukocyte antigen)	DQ883222	Presents peptides of exogenous proteins to CD4^+^ helper T cells and regulates humoral response.	gtgtgcgacggaatctataac (F)	60	86.8	83	258 (332–589)
			gagcatgagccctaagagac (R)				
Ii (invariant chain)	AB116558	Promotes proper folding and assembly of class II α/β heterodimers, facilitates intracellular transport of class II molecules, and segregates MHC class II exogenous antigen presentation pathway from the MHC class I antigen presentation pathway.	gcaacgccaccaagtacgg (F)	60	86.6	84	185 (332–516)
			aagagccactgacgcagcc (R)				
CD40	AF248545	Supplies a second antigen-independent signal (costimulation). Activates and regulates T cell immunity.	tcaagcagatggcgacagag (F)	60	85.6	82	198 (392–589)
			caccagggctctcatccga (R)				
CD80	AB026121	Supplies a second antigen-independent signal (costimulation). Activates and regulates T cell immunity.	agcgggagagagggtcttat (F)	60	83.0	79	123 (344–436)
			aagggcagtaatactaggcac (R)				
CD86	L76099	Supplies a second antigen-independent signal (costimulation). Activates and regulates T cell immunity.	gttcctatccaccagatgagt (F)	60	81.6	78	257 (343–599)
			gaagagacaccctgattgatac(R)				
IFN-α	M28623	Exerts potent antitumor, anti-viral, and immunomodulatory activities.	tgggagatcgtcagggcag (F)	60	83.9	81	155 (603–757)
			tgacatggcagaacaggagg (R)				
IFN-β	EF104599	Exerts potent antitumor, anti-viral, and immunomodulatory activities.	ggacagttgcctgggactc (F)	60	82.1	79	128 (127–254)
			tggagcatctcgtggataatc (R)				
GAPDH (glyceraldehde-3-phosphate dehydrogenase)	AF017079	Housekeeping gene used as an internal control in quantitative real-time PCR assays.	tggtgaaggtcggagtgaac (F)	60	86.4	83	225 (343–567)
			ggaagatggtgatgggatttc (R)				
5′-UTR of CSFV genome	AF531433	Regulates translation of the viral genomes.	gccatgcccatagtaggact (F)	60	86.5	83	116 (97–212)
			gcttctgctcacgtcgaact (R)				

Notes: F, forward; R, reverse.

Several previous studies have found that CSFV can evade the immune system and establish chronic infection [17,26,32]. More recently, global transcriptional profiles have been determined through microarray analysis in PBMC during CSFV infection *in vivo*[34]. These preliminary studies showed that certain immune response genes, SLA-2 (MHC class I), TAP1, SLA-DR (MHC class II), Ii, CD40, CD80, and CD86, were down-regulated *in vivo,* which correlates well with our *in vitro* data.

Recently, several studies have reported genomic expression profiles of macrophages, peripheral blood leukocytes, and peripheral blood mononuclear cells of pigs infected with a highly virulent strain of CSFV [34,36,37]. Graham *et al.* have characterized the cytokine response of peripheral blood cells of pigs following vaccination or infection with CSFV [38]. These published data showed transcriptional profiles similar to those in our study using peripheral blood cells during CSFV infection, and most of the immune response genes involved in CSFV infection *in vivo*. The *in vitro* gene expression patterns of these genes, as determined by real-time RT-PCR, was relatively consistent with that observed in macrophages and PBMCs *in vivo* during CSFV infection [34-36].

In the present study, transcription of nine immune response genes were analyzed. Interestingly, we observed that the mRNA levels of SLA-2, SLA-DR, and Ii genes changed during the early stages of CSFV infection, even before the CSFV RNA was synthesized (Figure 1). This indicates that the down-regulation of these genes could be due to interaction between CSFV and receptors on PK-15 cells that activate downstream pathways. It will be interesting in the future to investigate and identify which receptors are bound and activated by CSFV. We also showed that the mRNA levels of TAP1 and CD80 at 12 h and CD86 at 24 h decreased significantly at these later time points after CSFV infection with 0.1 and 1.0 MOI; CSFV began to transcript and to translate in PK-15 cells at 12 hpi, thus demonstrating that down-regulation of these particular genes may be related to CSFV replication. Collectively, these data suggest that CSFV infection is involved in inhibiting host immune response gene expression *in vitro,* both during entry into the cell as well as during replication. Furthermore, we have confirmed the accuracy of the transcriptional data obtained by real-time RT-PCR by showing that transcription of both SLA-DR and CD40 were consistent with protein levels measured by Western blot analysis of CSFV-infected PK-15 cells (Figures 3 and 4).

Finally, this study indicates that CSFV is involved in inhibiting expression of immune response genes, such as the MHC class II molecule SLA-DR, which function in antigen presentation. The mechanism by which CSFV interferes with MHC and how this helps the virus evade the host immune system is currently unclear. To further address and understand this mechanism, research into the regulation between CSFV and SLA-DR is in progress.

## Conclusion

In brief, this study has demonstrated that *in vitro* infection with classical swine fever virus inhibits the transcription of host immune response genes. These findings may facilitate the development of effective strategies for controlling CSF in China, and elsewhere in the World.

## Materials and methods

### Cells and virus

Porcine kidney (PK-15) cells, free of contaminating bovine viral diarrhea virus (BVDV), were used in this study. The cells were cultured in Dulbecco’s modified Eagle’s medium (DMEM) (Hyclone) with 10% heat-inactivated (56°C, 30 min) fetal calf serum (FCS) (Hyclone) and maintained at 37°C in a humidified environment containing 5% CO_2_ throughout the experiment. Seventy-two hours after culturing, the medium was replaced with fresh maintenance medium containing 1% FCS.

The highly virulent Shimen CSFV strain derived from China in 1945 was used in this study. A batch of virus stock (stored at −80°C) was propagated in PK-15 cell cultures. Viral infectivity was detected by an indirect immunofluorescence assay (IFA) using monoclonal antibody (LOM01, Jeno Biotch Inc.) against CSFV glycoprotein E2. CSFV titers were calculated using the method described by Reed and Muench.

### RNA extraction, cDNA synthesis and sequencing

Total RNA was extracted from samples using Trizol reagent (Invitrogen, Carlsbad, CA, U.S.) according to the manufacturer’s instructions and then digested with DNase I at 37°C for 15 min to remove contaminating DNA. The dried RNA pellets were resuspended in 50 μL of diethyl-pyrocarbonate-treated water. The concentration and purity of the total RNA were determined spectrophotometrically and by electrophoresis, respectively. The total RNA was used immediately, or stored at −80°C until being used for cDNA synthesis. First-strand cDNA was synthesized using Oligo dT_18_ primers and Superscript II reverse transcriptase (Invitrogen) according to the manufacturer’s protocol. Synthesized cDNA was stored at −80°C until use. The RT-PCR products of target genes were T/A cloned into pMD18-T vector (TaKaRa, Dalian, China) and then sequenced.

### Cloning and sequencing of target genes and confirmation of primer specificity

Total RNA was extracted from samples using the TRIZOL reagent and used as the template for first-strand cDNA synthesis using M-MLV reverse transcriptase with oligo dT_18_ primer. PCR was conducted using specific primers for amplification of the target genes. RT-PCR products were T/A cloned into pMD18-T vector and then sequenced. GenBank BLAST search revealed that the resulting nucleotide sequences of the target genes were identical to those published.

By using fluorescence detection, real-time RT-PCR offers continuous monitoring of the PCR reaction cycle-by-cycle. Detection in this assay is based on the binding of the fluorescence dye SYBR Green I, which binds all double-stranded DNA molecules. Because this process is sequence-independent, the characteristic melting temperature (T_m_) of the target is determined by performing a melting curve analysis on the PCR products to ascertain the formation of primer-dimers and non-specific products, which compete with the formation of specific PCR products. The PCR reactions were confirmed by both melting curve and gel analyses. In addition, melting curve analysis demonstrated that each of the primer pairs (Table 1) amplified a single, major product with a distinct T_m_ (Additional file 1: Figure S 1). No primer-dimers were generated in 40 cycles of real-time RT-PCR amplification.

### Quantitative real-time RT-PCR using SYBR Green I

The amplification dynamic curves, standard curves for each immune response gene, 5′-UTR of CSFV genome and GAPDH, were obtained using standard plasmids encoding the PCR products. The quantitative nature of the assay is demonstrated by the linear relationship between the log of the template copy number and the Ct value; a range with a correlation value (R) >0.99 is described. Melting curve analysis revealed that PCR amplified a single desired product. GAPDH was used as a housekeeping gene to correct for the amounts of total undegraded RNA in all samples. Expression of GAPDH was examined using real-time RT-PCR. Results showed that GAPDH mRNA levels did not vary between mock- and CSFV-infected cells, and the Ct parameter of GAPDH was found to vary between 12.24 and 12.95 (data not shown). The amplification efficiency of CSFV-specific cDNA was evaluated by comparing the standard curves. The standard curve for cDNA of the 5′-UTR of the CSFV genome was y= −3.295x+5.068, with a correlation coefficient r^2^=0.998, and standard curve showed a 7-log dynamic range of amplification. The high correlation coefficient and large dynamic range indicate that the amplification efficiency is reproducible and therefore could be used to quantify CSFV-specific RNA in PK-15 cell cultures (Additional file 1: Figure S 1).

Primer Premier software (PREMIER Biosoft International, U.S.) was used to design specific primers for several immune response genes (including SLA-2, TAP1, SLA-DR, Ii, CD40, CD80, CD86, IFN-α, and IFN-β), 5′-UTR of CSFV, and the housekeeping gene GAPDH based on known swine sequences (Table 1). Real-time RT-PCR was performed on the MJ Opticon II real-time PCR system (MJ Research, Inc) or the iCycler iQ real-time PCR detection system (Bio-Rad). Reactions were performed in a 25 μL reaction mixture containing 12.5 μL of 2× SYBR Green I PCR Master Mix (Tiangen Biotech, Beijing, China), 10 μL of either diluted cDNA or plasmid standard, 0.2 μL of each primer (25 pmol/μL), and 2.3 μL of ddH_2_O. The thermal cycling conditions included an initial heat-denaturing step at 94°C for 3 min, 40 cycles at 94°C for 20 s, 60°C for 20 s, and 68°C for 20 s, followed by a final extension step at 68°C for 5 min. A melting curve analysis was performed to assess primer specificity and product quality by denaturation at 95°C, annealing at 65°C, and melting at a rate of 0.1°C/sec to 95°C.

### Construction of the standard curve for the target genes

To determine the copy number of the target transcript, the cloned plasmid DNA for the target gene was used to generate a standard curve. Plasmids contained the cDNA inserts encoding the respective PCR products in a pMD18-T vector. The cloned plasmid DNA was diluted consecutively 10-fold at a range of 10^2^–10^9^ copies.

### Determination of target gene mRNA level by real-time RT-PCR

Determination of target gene mRNA level was similar to the standard curve. Each cDNA from the sample was run in triplicate and the mean value was calculated. The threshold cycle (Ct) values were averaged from each reaction. GAPDH was used as a reference to normalize mRNA levels.

### Detection of protein expression by Western blot

For Western blot analysis, specific antibodies were used: anti-His monoclonal antibodies (AB102-01) were purchased from Beijing TianGen Co. Ltd; specific SLA-DR monoclonal antibodies (MA1-19146) were purchased from Affinity BioReagents^™^ Co. Ltd; anti-CD40 monoclonal antibodies (ab13545) were purchased from Hong Kong Abcam Ltd; the monoclonal antibody for β-tubulin was purchased from Sigma Co. Ltd; horseradish peroxidase (HRP)-labeled goat anti-mice IgG (Sigma) or alkaline phosphatase house anti-mouse IgG (ZSGB-BIO) was used as the secondary antibody. The mock- and CSFV-infected PK-15 cells were collected by directly scraping the cells into the medium, rinsing once with cold PBS, transferring to a 1.5-ml Eppendorf tube, and lysing in cell lysate buffer (1% Nonide P-40, 0.5% sodium deoxycholate, 0.1% SDS, 5 mM EDTA, and protease inhibitor mixture). Samples were kept on ice for 1 h and then treated at 95°C for 5 min. The insoluble material was pelleted at 12,000 rpm for 5 min and stored at −80°C until use. Sodium dodecylsulfate-polyacrylamide gel electrophoresis (SDS-PAGE) was performed on 12% acrylamide gel in the Mini Protein Tetra system (Bio-Rad). Protein samples were separated by SDS-PAGE and then electroblotted onto an Immobilon-P^R^ Transfer (MILLIPORE) membrane using a Semi-Dry Transfer System (Bio-Rad) for 90 min. Non-specific proteins were blocked by incubating the membrane in TBS containing 0.05% Tween-20 (TBS-T) and 5% skimmed milk at 4°C. They were washed three times with TBS-T and then incubated with primary monoclonal antibodies for 90 min at 37°C. The membrane was washed three times with TBS-T and incubated subsequently with secondary antibody (alkaline phosphatase-conjugated horse anti-mouse IgG) for 60 min at room temperature. The specific protein bands were visualized by using the BCIP/NBT kit (TianGen Corp. Ltd.).

### Statistical analysis

For real-time RT-PCR, the Ct parameter was defined as the number of fractional cycles at which the fluorescence passed a fixed threshold above baseline. The Ct was taken from each reaction and converted to a relative amount by comparing it to a standard curve made from a serial dilution of plasmid. The relative amount of each transcript was normalized to the GAPDH control in the respective sample to determine the relative expression level of each particular gene. In order to show a clear trend in gene expression over the 48-hour time course of infection, the expression level of each gene of a mock-infected group at the tested time points was designated hypothetically as 1× and the change of infected groups was normalized accordingly.

Statistical analysis of the mRNA levels was performed using the SPSS11.5 for Windows statistical software package (SPSS Inc., U.S.). Further analysis was performed for samples producing a significant value (P<0.05) by one-way analysis of variance (ANOVA). All data showed a normal distribution and passed equal variance testing. Differences between means were assessed by the Tukey’s honestly significant difference test of *post hoc* multiple comparisons. Data are expressed as mean ± SD, and differences are considered significant at P<0.05.

## Competing interests

All authors declare that they have no competing interests.

## Authors’ contributions

TRL and LF were responsible for the research design, detecting transcriptional changes of immune response genes and paper writing. JL, XMM, HYZ, JJL, HL, SKS, XBC, LJS, SY, YSL, and XNL carried out the experiments. All authors read and approved the final manuscript.

## Supplementary Material

Additional file 1 **Figure S1.**Real-time RT-PCR for quantification of IFN-α gene mRNA. The mRNA levels of SLA-2, TAP1, SLA-DR, Ii, CD40, CD80, CD86, IFN-α, IFN-β, and GAPDH genes in PK-15 cells stimulated by CSFV were quantified by real-time RT-PCR. The real-time PCR amplification (a) dynamic curves and (b) standard curves were obtained by plotting fluorescence data against their cycle number. (c) represents the melting curves of different genes. Click here for file

## References

[B1] Moennig V The hog cholera virus Comp Immunol Microbiol Infect Dis 1992 15 189 201 10.1016/0147-9571(92)90092-6 1325334

[B2] Pearson JE Hog cholera diagnostic techniques Comp Immunol Microbiol Infect Dis 1992 15 213 219 10.1016/0147-9571(92)90094-8 1325335

[B3] Meyer H Liess B Hermanns W Diaplazentare Infektion von Schweinefeten mit dem Virus der Europäischen Schweinepest (ESP). Virologische und serologische Untersuchungen in der postnatalen Phase. Fortschritte der Veterinärmedizin Suppl Zbl Vet Med 1980 30 140 144

[B4] Edwards S Fukusho A Lefevre PC Lipowski A Peisak Z Roehe P Westerqaard J Classical swine fever the global situation Vet Microbiol 2000 73 103 119 10.1016/S0378-1135(00)00138-3 10785321

[B5] Wengler G Bradley DW Collett MS Heinz FX Schlesinger RW Strauss JH Murphy FA, Fauquet CM, Bishop DHL, Ghabrial SA, Jarvis AM, Martelli GP, Mayo MA, Summers MD Virus taxonomy Sixth Report of the International Committee on Taxonomy of Viruses Family Flaviviridae 1995 Springer-Verlag, 415 427

[B6] Fauquet CM Mayo MA Maniloff J Desselberger U Ball LA In the 8th report of the international committee on taxonomy of viruses Virus taxonomy 2005 Academic Press, Elsevier

[B7] Meyers G Rumenapf T Thiel HJ Molecular cloning and nucleotide sequence of the genome of hog cholera virus Virology 1989 171 555 567 10.1016/0042-6822(89)90625-9 2763466

[B8] Moormann RJ Warmerdam PA van der Meer B Schaaper WM Wensvoort G Hulst MM Molecular cloning and nucleotide sequence of hog cholera virus strain Brescia and mapping of the genomic region encoding envelope protein E1 Virology 1990 177 184 198 10.1016/0042-6822(90)90472-4 2162104

[B9] Floegel G Wehrend A Depner KR Fritzemeier J Waberski D Moenniq V Detection of classical swine fever virus in semen of infected boars Vet Microbiol 2000 77 109 116 10.1016/S0378-1135(00)00267-4 11042404

[B10] Choi C Chae C Localization of classical swine fever virus in male gonads during subclinical infection J Gen Virol 2002 83 2717 2721 12388807 10.1099/0022-1317-83-11-2717

[B11] Carrasco CP Rigden RC Vincent E Balmelli C Ceppi M Bauhofer O Tâche V Hjertner B McNeilly F Interaction of classical swine fever virus with dendritic cells J Gen Virol 2004 85 1633 1641 10.1099/vir.0.19716-0 15166448

[B12] Chen LJ Dong XY Shen HY Zhao MQ Ju CM Yi L Zhang XT Kang YM Chen JD Classical swine fever virus suppresses maturation and modulates functions of monocyte-derived dendritic cells without activating nuclear factor kappa B Res Vet Sci 2012 93 1 529 37 10.1016/j.rvsc.2011.06.026 21764089

[B13] Bensaude E Turner JLE Wakeley PR Sweetman DV Pardieu C Drew TW Wileman T Powell PP Classical swine fever virus induces proinflammatory cytokines and tissue factor expression and inhibits apoptosis and interferon synthesis during the establishment of long-term infection of porcine vascular endothelia cells J Gen Virol 2004 85 1029 1037 10.1099/vir.0.19637-0 15039545

[B14] Charleston B Fray MD Baigent S Carr BV Morrison WI Establishment of persistent infection with non-cytopathic bovine viral diarrhea virus in cattle is associated with a failure to induce type I interferon J Gen Virol 2001 82 1893 1897 11457995 10.1099/0022-1317-82-8-1893

[B15] Schweizer M Peterhans E Noncytopathic bovine viral diarrhea virus inhibits double-stranded RNA-induced apoptosis and interferons synthesis J Virol 2001 75 10 4692 4698 10.1128/JVI.75.10.4692-4698.2001 11312340 PMC114223

[B16] Susa M König M Saalmüller A Reddehase MJ Thiel HJ Pathogenesis of classical swine fever: B-lymphocyte deficiency caused by hog cholera virus J Virol 1992 66 1171 1176 1731095 10.1128/jvi.66.2.1171-1175.1992 PMC240821

[B17] Summerfield A Knötig SM McCullough KC Lymphocyte apoptosis during classical swine fever: implication of activation-induced cell death J Virol 1998 72 1853 1861 9499036 10.1128/jvi.72.3.1853-1861.1998 PMC109475

[B18] Markowska-Daniel I Pejsak Z Winnicka A Collins RA Phenotypic analysis of peripheral leukocytes in piglets infected with classical swine fever virus Res Vet Sci 1999 67 53 57 10.1053/rvsc.1998.0278 10425241

[B19] Lee WC Wang CS Chien MS Virus antigen expression and alterations in peripheral blood mononuclear cell subpopulations after classical swine fever virus infection Vet Microbiol 1999 67 1 17 29 10.1016/S0378-1135(99)00029-2 10392774

[B20] Summerfield A Zingle K Inumaru S McCullough KC Induction of apoptosis in bone marrow neutrophil-lineage cells by classical swine fever virus J Gen Virol 2001 82 1309 1318 11369874 10.1099/0022-1317-82-6-1309

[B21] Van Oirschot JT De Jang D Huffels ND Effect of infections with Swine fever virus on immune functions. II. Lymphocyte response to mitogens and enumeration of lymphocyte subpopulations Vet Microbiol 1983 8 1 81 95 10.1016/0378-1135(83)90021-4 6845636

[B22] Pauly T König M Thiel HJ Saalmuller A Infection with classical swine fever: effects on phenotype and immune responsiveness of porcine T-lymphocytes J Gen Virol 1998 79 31 40 9460919 10.1099/0022-1317-79-1-31

[B23] Cheville NF Mengeling WL The pathogenesis of chronic hog cholera (swine fever). Histologic, immunofluorescent and electron microscopic studies Lab Invest 1969 20 261 274 5773219

[B24] Ressang AA Studies on the pathogenesis of hog cholera. II. Virus distribution in tissue and the morphology of the immune response Zentbl Veterinarmed B 1973 20 4 272 288 4751662

[B25] Gomez-Villamandos JC Salguero FJ Ruiz-Villamor E Sanchez-Cordon PJ Bautista MJ Sierra MA Classical swine fever: pathology of bonemarrow Vet Pathol 2003 40 157 163 10.1354/vp.40-2-157 12637755

[B26] Sánchez-Cordón PJ Romanini S Salguero FJ Nunez A Bautista MJ Jover A Gomez-Villamos JC Apoptosis of thymocytes related to cytokine expression in experimental classical swine fever J Comp Pathol 2002 127 4 239 248 10.1053/jcpa.2002.0587 12443731

[B27] Summerfield A Knötig SM Tschudin R McCullough KC Pathogenesis of granulocytopenia and bone marrow atrophy during classical swine fever involves apoptosis and necrosis of uninfected cells Virology 2000 272 50 60 10.1006/viro.2000.0361 10873748

[B28] Summerfield A Hofmann M McCullough KC Immature granulocytic cells dominate the peripheral blood during classical swine fever and are targets for virus infection Vet Immunol Immunopathol 1998 63 289 301 10.1016/S0165-2427(98)00108-1 9656461

[B29] Gisler AC Nardi NB Nonnig RB Oliveira LG Roehe PM Classical swine fever virus in plasma and peripheral blood mononuclear cells of acutely infected swine Zentralbl Veterinaermed B 1999 46 585 593 10.1046/j.1439-0450.1999.00286.x 10605368

[B30] Summerfield A Hofmann MA McCullough KC Low density blood granulocytic cells induced during classical swine fever are targets for virus infecion Vet Immunol Immunopathol 1998 63 289 301 10.1016/S0165-2427(98)00108-1 9656461

[B31] Sato M Mikami O Kobayashi M Nakajima Y Apoptosis in the lymphatic organs of piglets inoculated with classical swine fever virus Vet Microbiol 2000 75 1 1 9 10.1016/S0378-1135(00)00198-X 10865147

[B32] Choi C Hwang KK Chae C Classical swine fever virus induces tumor necrosis factor-alpha and lymphocyte apoptosis Arch Virol 2004 149 5 875 889 10.1007/s00705-003-0275-6 15098104

[B33] Sánchez-Cordón PJ Núñez A Salguero FJ Pedrera M Fernandez de Marco M Gomez-Villamandos JC Lymphocyte Apoptosis and Thrombocytopenia in Spleen during Classical Swine Fever: Role of Macrophages and Cytokines Vet Pathol 2005 42 4 477 488 10.1354/vp.42-4-477 16006607

[B34] Li J Yu YJ Feng L Cai XB Tang HB Sun SK Zhang HY Liang JJ Luo TR Global transcriptional profiles in peripheral blood mononuclear cell during classical swine fever virus infection Virus Res 2010 148 1–2 60 70 20034523 10.1016/j.virusres.2009.12.004

[B35] Van Oirschot JT Leiss B Description of the virus infection Classical Swine Fever and Related Viral Infections 1997 Martinus Nijhoff, Boston 1 25

[B36] Borca MV Gudmundsdottir I Fernandez-Sainz IJ Holinka LG Risatti GR Patterns of cellular gene expression in swine macrophages infected with highly virulent classical swine fever virus strain Brescia Virus Res 2008 138 1–2 89 96 18796318 10.1016/j.virusres.2008.08.009

[B37] Shi Z Sun J Guo H Tu C Genomic expression profiling of peripheral blood leukocytes of pigs infected with highly virulent classical swine fever virus strain Shimen J Gen Virol 2009 90 Pt7 1670 1680 19264604 10.1099/vir.0.009415-0

[B38] Graham SP Everett HE Johns HL Haines FJ La Rocca SA Khatri M Wright IK Drew T Crooke R Characterisation of virus-specific peripheral blood cell cytokine responses following vaccination or infection with classical swine fever viruses Vet Microbiol 2010 142 1–2 34 40 19854006 10.1016/j.vetmic.2009.09.040

