# Supplementary material for: In vitro infection with classical swine fever virus inhibits the transcription of immune response genes
Source: Virol J. 2012 Aug 28;9:175. doi: 10.1186/1743-422X-9-175 (PMC3463435; doi:10.1186/1743-422X-9-175)
Supplement: Additional file 1 — Figure S1.Real-time RT-PCR for quantification of IFN-α gene mRNA. The mRNA levels of SLA-2, TAP1, SLA-DR, Ii, CD40, CD80, CD86, IFN-α, IFN-β, and GAPDH genes in PK-15 cells stimulated by CSFV were quantified by real-time RT-PCR. The real-time PCR amplification (a) dynamic curves and (b) standard curves were obtained by plotting fluorescence data against their cycle number. (c) represents the melting curves of different genes. [file 1743-422X-9-175-S1.doc]

**Figure S1 Real-time RT-PCR for quantification of IFN-α gene mRNA**

SLA-2 TAP1

**a
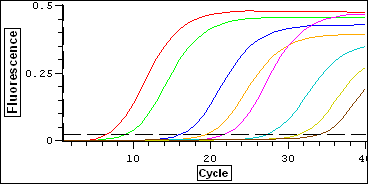
**
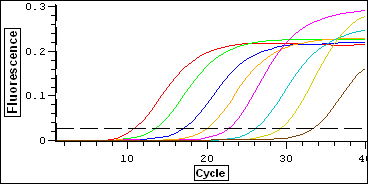


**b**
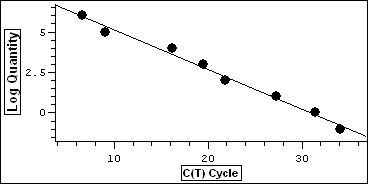
 **
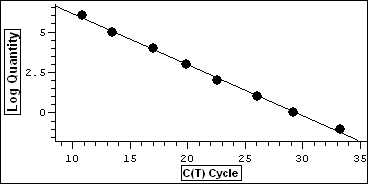
**

**c
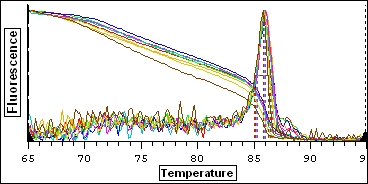

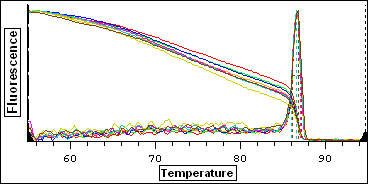
**

SLA-DR Ii

a
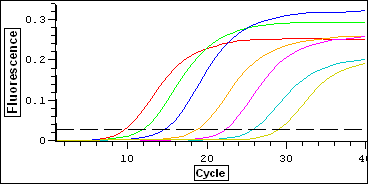

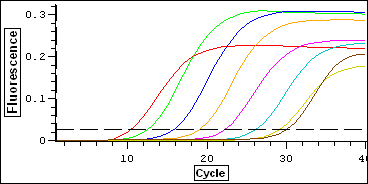


**b
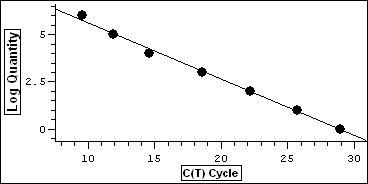

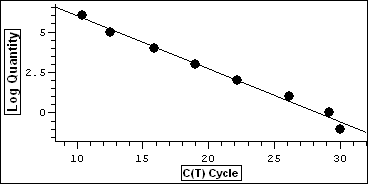
**

c
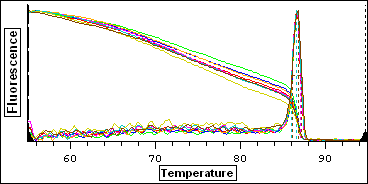

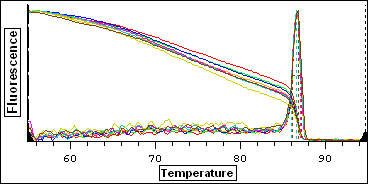


CD40 CD80

a
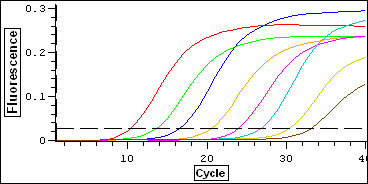

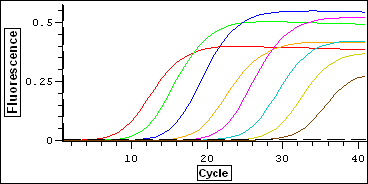


b **
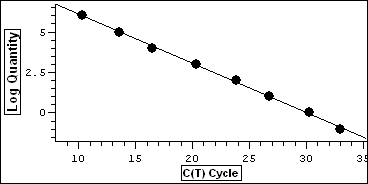
**
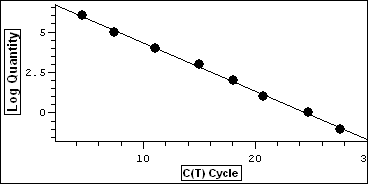


c
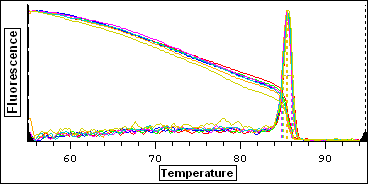

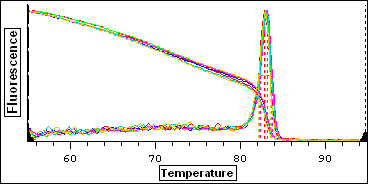


CD86 IFN-α

a
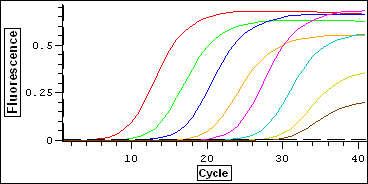

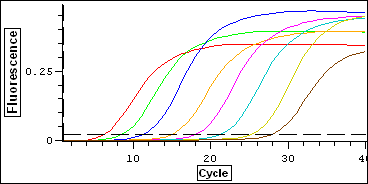


b
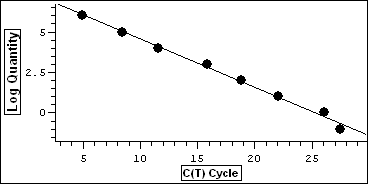

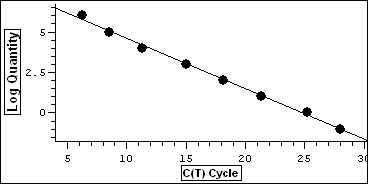


c
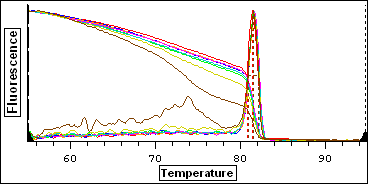

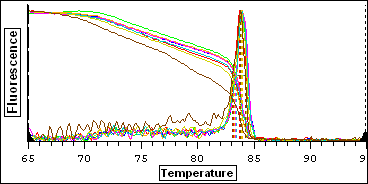


IFN-β GAPDH

a
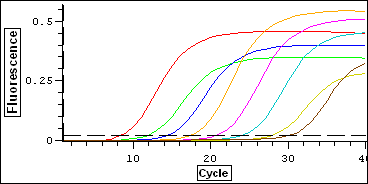

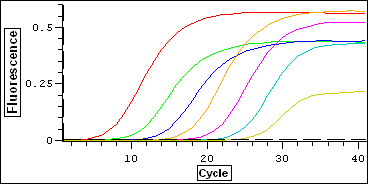


b
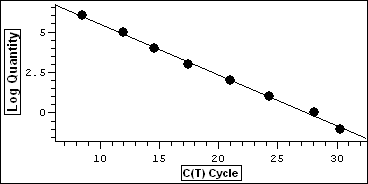
 **
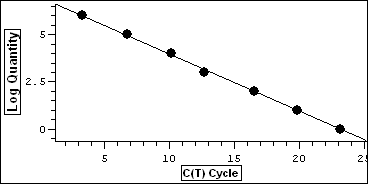
**

c
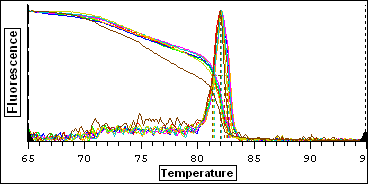
 **
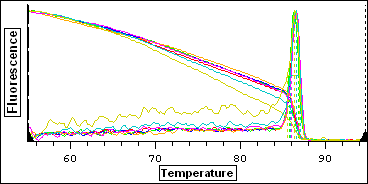
**
